# Recommendations for Standardizing Validation Procedures Assessing Physical Activity of Older Persons by Monitoring Body Postures and Movements

**DOI:** 10.3390/s140101267

**Published:** 2014-01-10

**Authors:** Ulrich Lindemann, Wiebren Zijlstra, Kamiar Aminian, Sebastien F.M. Chastin, Eling D. de Bruin, Jorunn L. Helbostad, Johannes B.J. Bussmann

**Affiliations:** 1 Department of Clinical Gerontology and Rehabilitation, Robert-Bosch-Hospital, Stuttgart 70376, Germany; 2 Institute of Movement and Sport Gerontology, German Sport University Cologne, Cologne 50933, Germany; E-Mail: zijlstra@dshs-koeln.de; 3 Laboratory of Movement Analysis and Measurement, Ecole Polytechnique Fédérale de Lausanne, Lausanne 1015, Switzerland; E-Mail: kamiar.aminian@epfl.ch; 4 School of Health and Life Science, Institute of Applied Health Research, Glasgow Caledonian University, Glasgow G4 0BA, Scotland, UK; E-Mail: Sebastien.chastin@gcu.ac.uk; 5 Institute of Human Movement Sciences and Sport, Department of Health Sciences and Technology, ETH Zurich, Zurich 8092, Switzerland; E-Mail: eling.debruin@hest.ethz.ch; 6 Department of Neuroscience, Faculty of Medicine, Norwegian University of Science and Technology, Trondheim 7491, Norway; E-Mail: jorunn.helbostad@ntnu.no; 7 Department of Rehabilitation Medicine, Erasmus MC University Medical Centre Rotterdam, Rotterdam 3015, The Netherlands; E-Mail: j.b.j.bussmann@erasmusmc.nl

**Keywords:** activity monitoring, older persons, physical activity, validation

## Abstract

Physical activity is an important determinant of health and well-being in older persons and contributes to their social participation and quality of life. Hence, assessment tools are needed to study this physical activity in free-living conditions. Wearable motion sensing technology is used to assess physical activity. However, there is a lack of harmonisation of validation protocols and applied statistics, which make it hard to compare available and future studies. Therefore, the aim of this paper is to formulate recommendations for assessing the validity of sensor-based activity monitoring in older persons with focus on the measurement of body postures and movements. Validation studies of body-worn devices providing parameters on body postures and movements were identified and summarized and an extensive inter-active process between authors resulted in recommendations about: information on the assessed persons, the technical system, and the analysis of relevant parameters of physical activity, based on a standardized and semi-structured protocol. The recommended protocols can be regarded as a first attempt to standardize validity studies in the area of monitoring physical activity.

## 1. Introduction

Physical activity (PA) is an important determinant of health and well-being. Physical inactivity is associated with mortality and loss of mobility [1,2] and contributes to the development of several chronic diseases [3]. Being regularly active substantially improves outcome and progression of most chronic degenerative diseases [4]. Additionally, an active life style contributes to social participation and quality of life, as expressed in the context of the “International Classification of Functioning, Disability, and Health” [5]. In order to understand how PA in daily life is associated with health and functioning in older persons, such behaviour needs to be studied in free-living conditions.

Contrary to young active adults, older persons perform most PA as part of every-day life activities related to work, house-holding and leisure time where energy cost is much lower than exercise, such as running. Thus, for many purposes, it is of more relevance to study aspects of PA in older persons, such as postural allocation and type of activity, than the energy expenditure associated with PA. Against this background, the World Health Organisation considers that PA can be measured by its four main components, which can be abbreviated as FITT: **F**requency of the activity (e.g., number of walking periods), **I**ntensity of the activity (e.g., walking speed); **T**ime or the duration of the bout of activity (e.g., duration of walking episodes), and the **T**ype of activity (e.g., lying, sitting, standing, walking) [6]. Where the FITT components apply to the population at large it is reasonable to expect that the weight of the individual FITT components will vary largely for different sub-population. Hence, the daily life performance of mobility related activities (such as standing or walking) can be considered as a key construct of PA in older people. However, since PA patterns differ so much between different populations, studies addressing PA should carefully define the key construct(s) which correspond to the specific topic and population under study.

The formal definition of PA is “any bodily movement produced by skeletal muscles that results in energy expenditure” [7]. This definition specifically focuses on the amount and volume of PA and the energy expenditure associated with PA, and thus, a large portion of PA literature has focused on the effect of physical exercise and on energy consumption, mostly from the perspective of health. However, besides activity related energy expenditure, PA is of interest in terms of body posture and movement behaviour.

Assessment of PA has traditionally been done by use of questionnaires, mostly focusing on leisure time levels of PA and on energy expenditure. Questionnaires have known limitations with respects to reliability and their relationship with actual behaviour [8,9], and they do not have the potential to assess all aspects of PA [10], especially in older persons [11]. Objective, performance-based laboratory tests will neither represent the usual performance of the tested individual [12]. A recent development is the use of wearable motion sensing technology which offers measurement in real-life conditions. Initially, raw acceleration signals from wearable sensors have been used to derive outcomes, such as activity intensity or energy expenditure of PA, but another option is to focus on human body postures and movement behaviour. Based on the use of miniaturised motion sensors, methods are currently available for long-term monitoring of body postures and movements under real-life conditions [13].

The detection of transitions and activities can be improved by considering human biomechanics or fuzzy rules [14]. These rules can considerably improve the classification performance and avoid false detection that biomechanically is inconsistent (e.g., leaning backward during standing), or improbable (e.g., a postural transition during walking). Machine learning and pattern recognition techniques can also improve the detection of transition and activity [15,16]. These approaches need generally true transitions and activities that can be used to learn a classification model or match a pattern of movement.

Most of the available sensor-based methods to assess body postures and movements have been applied in younger persons [17–20]. Only some of them have been validated in older persons [15,21–25]. Furthermore, most validation has been performed in in-lab settings, and there is at present little knowledge about whether laboratory results are transferable to real-life conditions [15].

In order to determine the degree to which a specific monitoring method is able to capture mobility related activities and postures of older people, such as described in FITT, specific validation studies in older persons are necessary. However, a major problem in interpreting the available validation studies of specific monitoring methods is the variety in validation protocols and applied statistics. This makes it hard to compare different monitoring methods, and it prevents researchers and other users in making justified decisions about the most appropriate method. We feel that the lack of guidelines and standardization plays an important role in this issue. Given the present rapid development of commercially available monitoring methods, this issue is gaining even more importance, since clinicians interested in monitoring aspects of PA are ill-advised when relying on producer information only.

The aim of this paper is to push the standardization of the methodology and therefore to formulate recommendations for assessing the validity of sensor-based activity monitoring aiming at determining PA with respect to body postures and movements of older persons. This project can be regarded as a first step in developing different validation protocols for different types of activity monitors and different populations.

## 2. Methods

Based on the systematic literature search of a recent review [26], validation studies of body-worn sensors to assess PA of older persons were identified. By personal communication additional articles were considered. To be included, studies had to focus on systems providing parameters on body postures and movements; studies calculating only general activity, such as activity counts were excluded since the interpretation of activity counts depends on the type of activity.

As a first step, the activity protocols and applied statistics of the included validation studies were summarized. From this summary, a first draft of the proposal statements was made, which was the start of an extensive inter-active process of writing, expert consulting and commenting, discussion, and rewriting. This first draft was sent to all authors, most of them authors of the included studies, who agreed to participate (*n* = 7). Their comments were processed in a second draft, which was discussed in a personal meeting held in Zurich in 2012.

**Figure 1.**
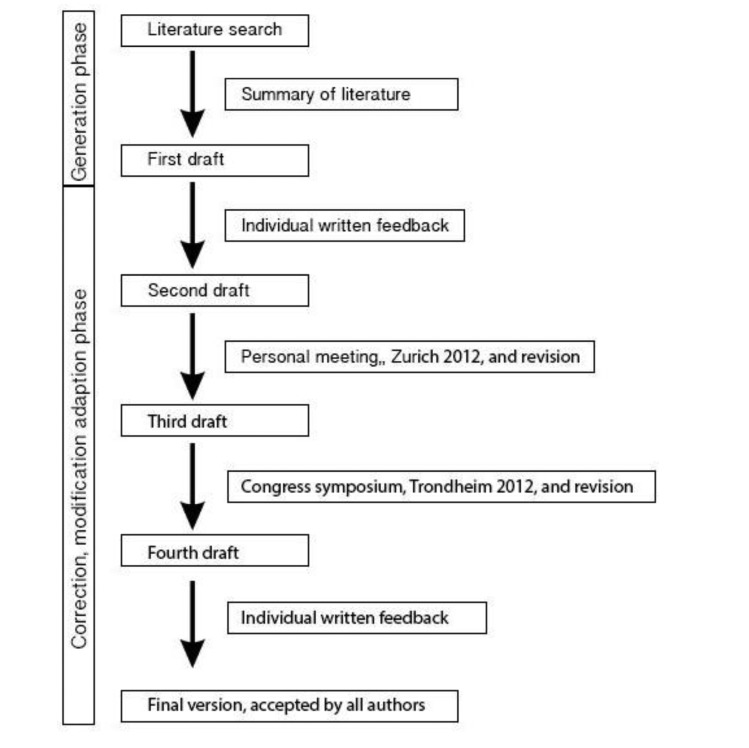
Description of the writing process.

The comments and conclusions from that meeting resulted in a third draft. The initiative and the third draft were presented and discussed in a symposium of the first Joint World Congress of the International Society for Posture & Gait Research/Gait & Mental Function (Trondheim, Norway, 24–28 June 2012,). The feedback of this symposium was included in the next draft that—after some additional expert consultation rounds—resulted in a final version that was accepted by all authors. The process of writing these recommendations is outlined in Figure 1.

## 3. Results/Recommendations

### 3.1. Persons to be Assessed

The general principle of validation studies is that subjects included must represent the target group. Older subjects are defined as being over 65 years of age, and therefore the persons to be included in validation studies must be aged 65 years or older. Persons in need of a walking aid should be part of a study group, because they represent one relevant sub-population within this group of over 65 years of age. A detailed description of the individuals must be provided, in terms of:

- general information on age, gender, weight, height
- co-morbidities
- use of walking aids
- gait speed
- description of gait abnormalities, such as asymmetric movement patterns (e.g., stroke)
- living situation (such as community, hospital, nursing home, *etc.*)

### 3.2. System Description

The sensor system that is validated should be clearly described in terms of:

- sensor specification (instruments included with range and sampling frequency)
- type, model, and series of both hard- and software
- hardware characteristics (weight, dimensions)
- wearing characteristics (location, fixation method)
- variables to be derived and algorithms, if available

Furthermore, a description of the underlying algorithms or an appropriate reference to a paper describing these algorithms has to be provided.

### 3.3. Relevant Characteristics of PA

As stated earlier, the outcome parameters to describe PA are related to body postures and movements, as characterized by the FITT principle. Starting from the type of parameters, main types of body postures and movements are formed by lying, sitting, standing, walking and body transitions (e.g., from sitting to standing position). For most applications in older persons this basic set of body postures and movements will be sufficient. However, it might be possible that more detailed information is required on sub-categories within this basic set, such as the type of walking (e.g., uphill, downhill, stairs, walking slow or fast), type of standing (e.g., quietly, or with some movement), and type of lying (e.g., back, side). It is also possible that special interest exists in other body postures and movements, such as cycling or driving a wheelchair. Once the type of activity is classified other features such as the duration of activity and its frequency over the day or week can be estimated.

### 3.4. Protocol

In order to evaluate the performance of activity monitors aiming at determining body postures and movements, standardization of the validation protocol is a pre-requisite. The authors propose a protocol that consists of different parts. Depending on the target scenario, a specific validation protocol may be created that includes parts of the protocol. The protocol consists of two main distinguishable parts: a standardized protocol, and a semi-structured protocol.

### 3.5. Standardized Protocol

The general ability of the instrument to detect different body postures and movements has to be assessed in a standardized protocol with a fixed order of instructions. The standardized protocol can be assessed in a laboratory setting. The added value of such a highly standardized protocol is to allow precise comparison of different monitoring methods and algorithms to detect highly relevant types of body postures and movements. In addition, standardized protocols may be used to study the effects of manipulating movement characteristics (e.g., walking speed and distance) on the outcomes of detection algorithms. The standardized protocol should include body postures and movements that are common, and that are known to be challenging for detection.

#### 3.5.1. Walking

With regard to the known problems of the general ability of detecting walking events at slow gait speed [21,27], different walking speeds must be tested in a separate protocol on a treadmill to identify a threshold where gait is recognized reliably. In order to clearly separate different walking speeds, the time period of each speed must be at least 30 s. Thresholds of gait speed that can be recognized by the activity monitor must be given in the results.

In combination with other body postures and movements and apart from treadmill testing, different distances of walking (for example 2, 5, and more (e.g., 10) meters) should be part of a standardized protocol with slow and normal gait speed, each. In addition, the protocol can include variations of walking, such as uphill and downhill walking and stair climbing (ascending, descending of four steps each).

#### 3.5.2. Body postures and movements other than walking

Other activities to be included are relevant body postures and movements other than walking, such as standing, sitting, and lying. Furthermore, posture transitions, such as sit-to-stand, stand-to-sit, sit-to-walk, walk-to-sit, lying-to-sit-to-stand, stand-to-sit-to-lying, lying-to-sit-to-walk, and walk-to-sit-to-lying have to be included. A unique order of body postures and movements (including transitions) is not necessary as long as all body postures and movements are performed at least once. The protocol can include body movement other than walking such as cycling, if appropriate for the investigated cohort.

### 3.6. Semi-Structured Protocol

The standardized protocol lacks ecological validity, because activities are not performed in a natural way and order, and thus cannot be used alone to validate spontaneous activity in real-life. Therefore, assessing (ecological) validity in real-life conditions is a second part of the proposed validation protocol. Ideally, the semi-structured protocol should be assessed in and around the persons' real home including a scenario of tasks that the subject can perform in an individual order. These tasks should correspond to the main daily activities that people naturally perform in the proposed environment. The activities should be relevant for older persons and should include several episodes of walking, standing, sitting, and lying. As an alternative to collecting validation data in the home environment, semi-structured protocol in a public environment, such as a hospital, or in one or several rooms in a laboratory setting is acceptable, as long as several general activities of daily living (for example filling a wash machine, cleaning windows, carrying a weight for some meters, floor sweeping, …) can be simulated.

Instructions during the semi-structured protocol should focus on the activity (there should be any kind of walking, standing, sitting, and lying), but not on the way how to perform this activity. The protocol should include relevant activities as mentioned above, so for the combination of all relevant activities duration of at least 30 min is estimated. This type of protocol is appropriate, if an overall measure of activity is the outcome parameter. Each body posture and movement should be clearly defined, such as walking being a cyclic movement of two or more events. Moreover, it has to be defined, if steps or strides are recognized as one event of walking.

### 3.7. Reference Criteria

As reference system to address the accuracy of the sensors to detect specific postures and movements a video observation can be regarded as the optimum method to assess concurrent validity, because of best reproducibility. At least two independent raters should do the classification of activities from the video. The mean results of the two independent persons should be regarded as reference outcome values and the difference as the reference error. Second choice of concurrent validation is direct observation by at least two observers with electronic classifiers (palm). Calculation of error between the two raters and the inter-observer reliability should be reported and be of acceptable quality in both cases.

Since existing PA monitors are validated against a reference criterion, they themselves cannot serve as the reference criterion for concurrent validity, but can serve as a reference criterion for construct validity, if they provide a maximum of performance for validation with sensitivity and specificity of at least 90% each. Other criteria such as number of sensors and power consumption can be neglected for the reference system, since the objective is to use this reference system only for validation purpose.

### 3.8. Analysis

Especially for the semi-structured protocol the evaluation of performance classification should include comparison of each body posture and movement episode, but not the overall activity (cumulative) during the measurement. Sensitivity, specificity, as well as positive and negative predictive values should be calculated with regard to the number of samples in each type of body posture and movement. With regard to the FITT concept, outcomes derived from the type of activity have to be evaluated, such as:

(1) the frequency of the episodes of different body postures and movements (e.g., number of walking episodes)
(2) the duration of the body postures and movements (e.g., cumulative walking time, longest walking episode)

There is no recommendation from our group with regard to intensity, because the focus here was on body postures and movement, which are more relevant outcomes in the older population with low intensity activities as performed during everyday life.

The number of transitions between body postures and movements need to be evaluated and the agreement should be also given in relation of the number of transitions. Consistency of classification should also be considered for validation. For example, if the same number of sit-stand and stand-sit transitions has been classified, this is a simple way to evaluate the consistency of the validation. The development of detection algorithms should be limited to an initial data-set with subsequent validation on data which was not used for development. The main results and recommendations are summarized in Table 1.

## 4. Discussion and Perspective

The recommended protocols can be regarded as a first attempt to standardize validity studies in the area of PA monitoring. It is a first step in several aspects. First of all, the current protocol is not defined and described in any detail. Further specification will be needed, but still the protocol can serve as the point of departure for following steps. Secondly, the protocol focuses on the application in older persons. Underlying choices are the population (older persons) and the focus on body posture and movement detection. With regard to FITT, where in younger age groups the emphasis should likely be put on the frequency and intensity of activities the emphasis for older age groups is better put on time and duration or type of activities. Application in other sub-populations; e.g., older people with chronic diseases, and with other outcomes will have to result in other validation protocols from which the results can be generalised to these sub-populations. Therefore, the defined recommendation can be seen as one tool in a general tool box of recommendations for activity monitoring. The recommended protocol comprises a standardized protocol in the laboratory allowing precise comparisons between devices, and a semi-structured protocol with free conditions reflecting real-life. The rationale for this protocol is the potential to target several measurement scenarios with different outcome measures. Since the assessment of PA in the persons' real-life environment ensures high ecological validity, this latter type of protocol is indispensable for assessment of real-life PA. In contrast, the validation protocol may be reduced, if the assignment of one activity monitor is reduced to the assessment of specific outcome parameters (e.g., duration upright position).

A widely accepted validation protocol will allow researchers and users to compare existing body-worn activity monitors, to identify their strengths and weaknesses with respect to defined activities, cohorts, and environments. Furthermore, the protocol could be used to validate future systems. Application of the protocol will surely identify those systems that are best dedicated for monitoring PA in older persons. Using the right system for a described cohort of older persons, PA databases for these cohorts can be established. These databases not only have to include the description of the cohort, such as the background information and the type of PA monitor as recommended, but also have to include other external parameters which may influence mobility patterns, such as the weather or season.

A limitation of the recommended protocol is that the issue of energy expenditure, reflecting intensity of PA, has not been included. This aspect was not included in the recommendations, because the focus here was on body postures and movements, which are more relevant outcomes in the older population with low intensity activities as performed during everyday life.

Future research may target to further develop the analysis of PA. Research activities may identify new parameters to describe PA. Furthermore, PA patterns, *i.e.*, sequences of different activities [28], may help to understand PA of different cohorts. In this context, further development of gait analysis during long term PA monitoring [29] may provide gait outcome parameters to predict future events, such as falls. Then additional validation procedures are needed. Similarly to quantitative and qualitative gait measures, other relevant activities, e.g., the sit-to-stand transfer, may be of interest. Although video recording can be used, there also would be the need for specific validation procedures. At last, there is a large push to share sensor data of PA assessments to enhance the development of new algorithms and for data aggregation to understand the determinants of PA and its relation to health. Cloud based solutions would allow researchers to share data, but this will require common, valid, and exhaustive data taxonomy [30].

## 5. Conclusion

The recommended protocols can be regarded as a first attempt to standardize validity studies in the area of monitoring PA of older persons. Application in sub-populations will have to result in specific validation protocols. A widely accepted validation protocol will allow comparing existing body-worn activity monitors and could be used to validate future systems. Future research may target to further develop the analysis of PA.

## Figures and Tables

**Table 1.** Main results and recommendations.

**A validation study of physical activity monitors must provide …**

information on the subjects included. a description of the system. a description of outcome body postures and movements (e.g., walking). a description of the underlying algorithms.

**The test protocol should consist of …**

a standardized protocol aiming at general ability of the instrument to detect different body postures and movements with a fixed order of instructions. a semi-structured protocol assessing validity in real-life conditions.

**As reference criteria …**

video observation can be regarded as the optimum method. second choice is direct observation by at least two observers.

**With regard to analysis …**

outcomes, such as the frequency of the episodes of different body postures and movements and the duration of the body postures and movements have to be evaluated. the evaluation of performance classification should include comparison of each body posture and movement episode. sensitivity, specificity, as well as positive and negative predictive values should be calculated with regard to the number of samples in each type of body posture and movement. the number of transitions between body postures and movements need to be evaluated. the development of detection algorithms should be limited to an initial data-set with subsequent validation on data which was not used for development.
